# Simple Orthodontic Correction of Rotated Malpositioned Teeth Using Sectional Wire and 2 × 4 Orthodontic Appliances in Mixed-Dentition: A Report of Two Cases

**DOI:** 10.1155/2020/6972196

**Published:** 2020-07-01

**Authors:** S. Nagarajan M. P. Sockalingam, Ahmad Shuhud Irfani Zakaria, Khairil Aznan Mohamed Khan, Fayyadhah Mohd Azmi, Nurhidayah Muhd Noor

**Affiliations:** Department of Family Oral Health, Faculty of Dentistry, The National University Malaysia (UKM), Malaysia

## Abstract

The correction of rotated malpositioned tooth/teeth into the dental arch alignment in the mixed-dentition is often a challenging task for paediatric dentists. Failure in addressing this issue can bring about detrimental effect to the developing dentition and increases the probability of a complex orthodontic treatment in later years. Factors such as severity of the malpositioned teeth, patient's treatment compliance, limitation in specific functions of the selected appliance, availability of bone and space may dictate the success of the treatment. The combined use of a simple sectional orthodontic wire appliance and a 2 × 4 orthodontic appliance has been shown to produce a positive effect. The appliances resulted in derotation of the rotated malpositioned teeth and bringing them into arch alignment in two cases. This treatment option had eliminated the detrimental effects to the developing dentition and helped patients to enhance their smile and dental aesthetics.

## 1. Introduction

The transition period from primary to permanent dentitions between 6 and 12 years of age is often a worrisome period for parents. Parents expect their children's permanent teeth to erupt into beautiful dental aesthetics following shedding of the primary teeth [1]. Even children are aware of dental aesthetic of their teeth and that of others [2]. However, nature's expressions often lead to imperfection of teeth alignment in some children.

Malocclusion of teeth in dentitions are either a malrelationship of dental arches or malalignment of teeth [3]. Malocclusion refers to a significant deviation from the perfect occlusion of teeth that is aesthetically unsatisfactory [4]. Malocclusions are categorised into skeletal or dental malocclusions, which are either hereditary or environmental in nature [5]. Malposition of teeth refers to altered positioning of one or more teeth from a well-aligned jaw [3]. Malposition of teeth are often caused by local factors such as presence of supernumerary tooth or teeth, odontomes, cysts, trauma to the primary tooth that displaces the permanent successor tooth germ, ectopic tooth germ and bony defect of the alveolus due to cleft lip and palate, or other localised bony pathologies [6].

Management of malpositioned teeth in the early stage of mixed dentition possesses great dilemma to dentists. The type of treatment depends on the severity of the malposition, availability of the occlusal space, availability of alveolar bone architecture, availability of eruption path, and patient's compliance for treatment. Simple discrepancies in the teeth alignment can be corrected with some simple methods such as use of a wooden spatula tongue blade, composite or glass ionomer cement incline plane, Catlan's appliance and removable appliance with z-spring, and expansion screw or micro screw [7–9]. However, correction of greatly rotated or malposition teeth away from the arch alignment requires slight improvisation in the alignment methods because the use of simple and removable appliances may not be effective in correcting the malpositioned teeth effectively.

This paper describes management of two cases of malpositioned and rotated permanent teeth in the premaxilla treated with simple sectional orthodontic wire appliance and followed by two-by-four (2 × 4) orthodontic appliance.

## 2. Case Report

### 2.1. Case 1

**Figure 1:**
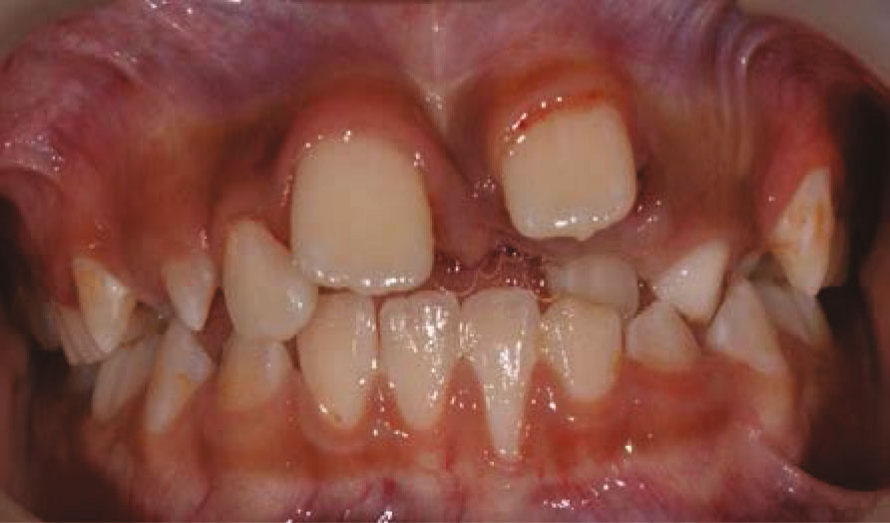
Pretreatment photograph showing wide diastema median, anteriorly positioned tooth 21, palatally malposition tooth 22, and gingiva recession of tooth 31 due to traumatic occlusion from the extracted supernumerary tooth.

A 10-year-old boy was referred for management of malposition left maxillary permanent lateral incisor (tooth 22). Tooth 22 was in crossbite to the left mandibular permanent lateral incisor (tooth 32) and permanent canine (tooth 33) (Figure 1). According to the boy's mother, the boy had a small pointed tooth between the right and left maxillary permanent central incisors (tooth 11 and tooth 21) extracted two months ago at a private dental clinic. The attending dentist informed them it was an additional tooth. However, no X-ray was available to verify their account. On examination, tooth 21 appeared malposition labial and inferior in relation to the maxillary alveolar ridge.

**Figure 2:**
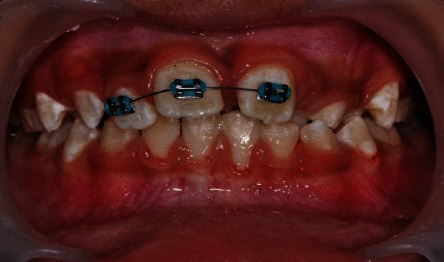
Sectional orthodontic wire appliance to guide tooth 21 into arch alignment through deflection technique.

The first stage of the treatment was to get tooth 21 closer to the arch alignment. We bonded orthodontic brackets (022 × 028 slots, American Orthodontics) on teeth 12, 11, and 21 and used a sectional orthodontic appliance technique to align the teeth. It took over 4 months to achieve the desired result (Figure 2). A 014 round nickel titanium (NiTi) wire was used to align tooth 22 through deflection method. Once tooth 21 had been moved inferiorly towards the arch alignment, we placed molar bands around the maxillary permanent first molars (teeth 16 and 26). However, the mesial-distal width space between tooth 21 and the left maxillary left primary canine (tooth 63) was narrow (6 mm) and inadequate to move tooth 22 into the arch alignment.

In the second stage, we used a 2 × 4 fixed orthodontic appliance to close the space between tooth 11 and tooth 21. We passed a 018 stainless steel round wire through the brackets and bands. We ligated both the brackets on teeth 12 and 11 over the arch wire using a ligature wire to act as an anchor unit. A 3-unit power chain was placed over the brackets on tooth 12, tooth 11, and tooth 21 to gain space closure. The space closure took approximately 2 months.

**Figure 3:**
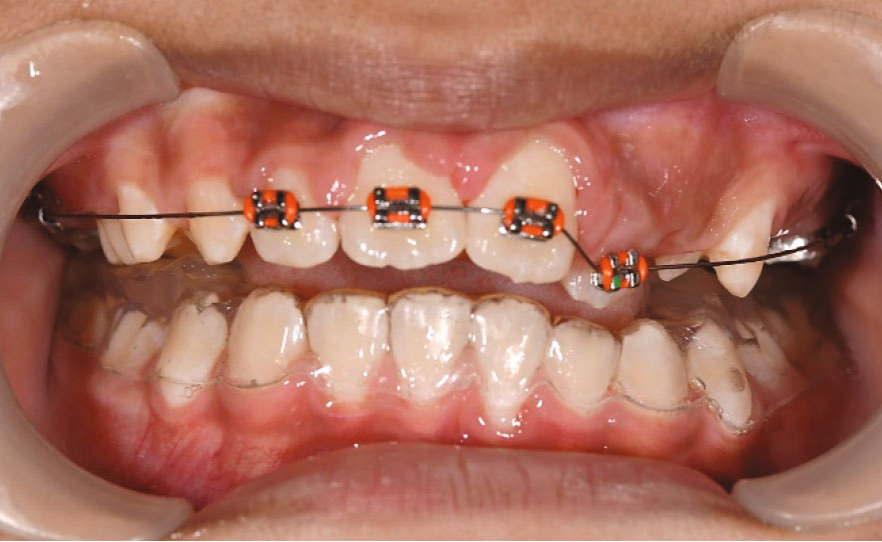
A 2 × 4 orthodontic appliance to guide malpositioned tooth 22 into arch alignment.

**Figure 4:**
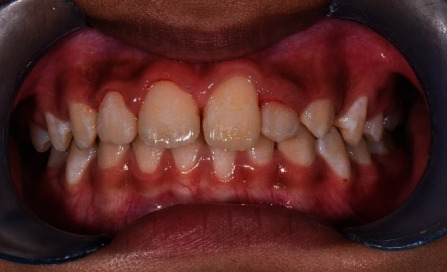
Tooth 21 and tooth 22 brought into acceptable arch alignment and the severity of gingiva recession of tooth 31 reducing.

The third stage is to get the malposition tooth 22 into the arch alignment. We constructed an Essix appliance over the mandibular teeth to raise the bite anteriorly for movement of teeth. Initially, we used a 012 round NiTi orthodontic arch wire to bring tooth 22 into alignment (Figure 3). Subsequently, we replaced the wire gradually to 014 and 016 round NiTi arch wires over the following months. After four months, we brought tooth 22 anteriorly over teeth 32 and 33 into alignment (Figure 4). A 19 × 25 stainless steel rectangular wire used for retention for 6 months.

### 2.2. Case 2

**Figure 5:**
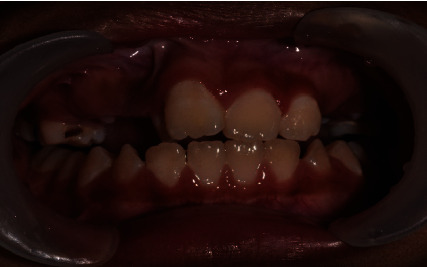
Malposition and rotate tooth 12 palatal to tooth 11.

A 9-year-old boy was referred from a private dental clinic for management of palatally placed tooth 12. On examination, tooth 12 was found to be palatal to tooth 11, rotated, and in a crossbite to the lower incisors (Figure 5). We placed orthodontic brackets (022 × 028 slots, American Orthodontics) on the four maxillary permanent incisors and molar bands around the maxillary permanent first molars for a 2 × 4 orthodontic appliance to align tooth 12. However, it was not possible to place a stable 012 round NiTi orthodontic wire through the bands and brackets due to a long span flexible archwire. This is mainly because of the absence of the right maxillary permanent premolars and primary molars.

**Figure 6:**
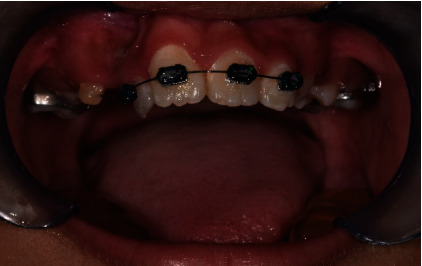
Sectional orthodontic wire appliance used to rotate and bring tooth 22 anteriorly into arch alignment.

**Figure 7:**
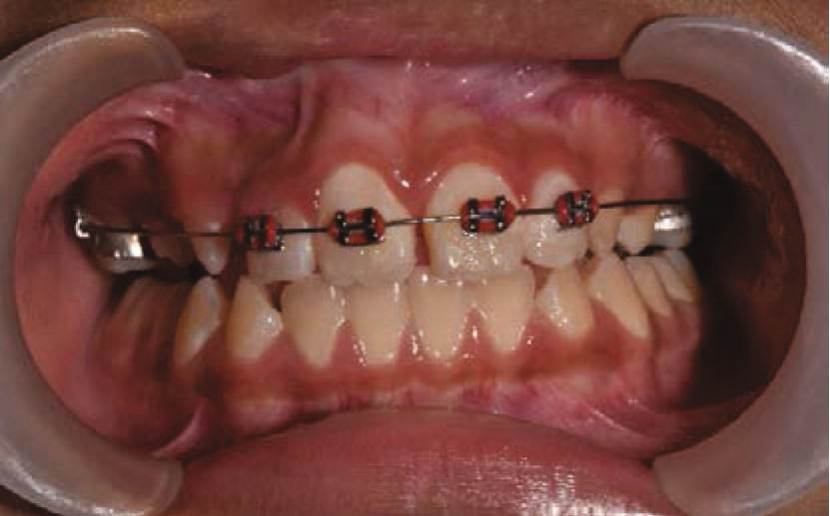
A 2 × 4 orthodontic appliance used to bring all permanent upper incisor into arch alignment.

**Figure 8:**
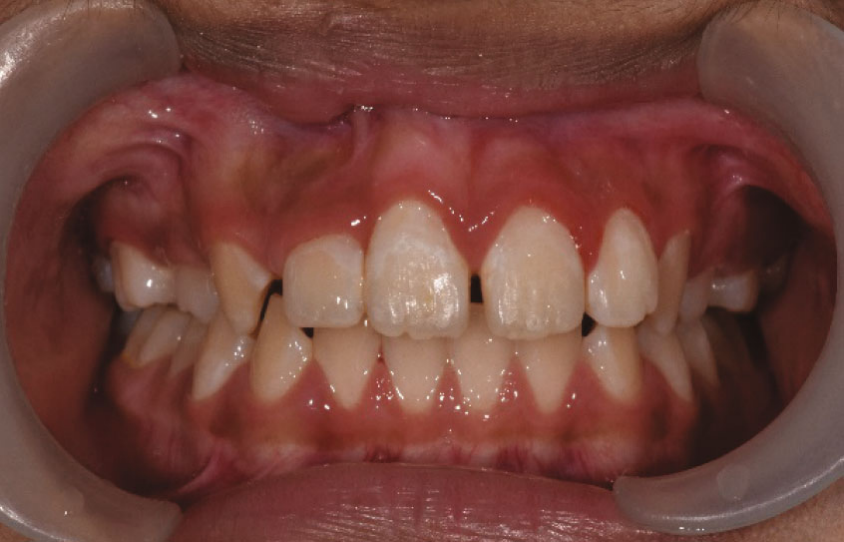
Six months posttreatment photograph: patient's upper incisors in an acceptable arch alignment.

At this stage, we decided to proceed with a sectional orthodontic wire appliance to initiate the treatment. We placed a sectional 012 round NiTi orthodontic wire on the brackets and held it in position with elastic ligature ties. The free end of the 012 NiTi wire on the right side was attached to the tip of the erupting right maxillary permanent canine (tooth 13) with composite resin (Figure 6). This provided an anterior pull effect of the wire on the rotated tooth 21. We also constructed an Essix appliance over the mandibular teeth to allow bite opening anteriorly. The sectional 012 round NiTi orthodontic wire was replaced with 014 NiTi and 016 NiTi wires, respectively, in the subsequent months. We replaced the sectional orthodontic wire appliance with the 2 × 4 orthodontic appliance once two-third of the crowns of the premolar and canine clinically seen (Figure 7). Finally, a 19 × 25 stainless steel rectangular wire was used as retention for 6 months. The process of bringing the rotated tooth 12 into arch alignment took us close to 7 months (Figure 8).

## 3. Discussion

Malposition teeth if left untreated lead to complications in the developing dentition with may be detrimental to either to the dental functions or the dental aesthetics [10]. Common problems encountered are issues related to loss of space due to migration of adjacent teeth into the available space [11]; traumatic bite on opposing teeth that lead to gum recession and mobility of teeth [12]; and attrition of enamel surface of the opposing teeth in contact [7]. Complications such deviation of the jaw and temporomandibular joint problems are common if premature contact on occluding teeth found [13]. Other issues include difficulty in maintaining oral hygiene such as brushing and flossing around the misaligned teeth that give rise to caries and gingivae problems [14].

Dentists face many challenges in treating malposition teeth. In young and growing patients, issues such as patient's treatment compliance, parental expectations, and selection of an appropriate appliance can be a problem. Dentists often leave the malposition teeth with no treatment until a later stage for orthodontics correction. However, orthodontic treatment at a later stage is often prolonged and complex because of severe space loss and migration of adjacent teeth. Opposing teeth may become mobile or loose because of gum recession by the time dentist start orthodontic treatment. In view of the delayed detrimental effects to the dentition, children in mixed dentition need orthodontic screening to identify issues related to malocclusion [15].

Achieving and ensuring perfect teeth alignment and aesthetics in a developing dentition are not the duties of paediatric dentists. The role of paediatric dentists is to minimise the detrimental effects caused by malocclusion in the developing dentition and facilitate an easy transition of care to orthodontists later [16]. Paediatric dentists and orthodontists should work hand in hand to streamline what is achievable during growing phases of the dentitions concerning interceptive orthodontics [17]. Knowledge on the mechanics of fixed orthodontic is useful for paediatric dentists. The knowledge will allow paediatric dentists to improvise treatment methods to address the challenges faced when the use of a removable appliance meets its limits.

In this article, we have highlighted two methods of teeth alignment: the sectional wire orthodontic appliance method and the 2 × 4 fixed orthodontic appliance method. The sectional orthodontic wires have been shown to correct minor to moderate anterior crossbite of teeth in a published article [18]. Children tolerated well with use of the sectional orthodontic wire method, and this method offers many advantages. The 2 × 4 orthodontic appliance also allows a well-controlled three-dimensional tooth movement [19, 20]; however, this appliance may not be effective if there are only a few teeth available in the dentition. Fewer teeth will not allow proper sitting of the long span arch wire, which may dangle and dislodged from its brackets or cause discomfort to patient.

In the described cases, short orthodontic wires were used to bring forward or de-rotate the malposition teeth during the initial stages of treatment, followed by 2 × 4 fixed orthodontic appliances to control tooth alignment and retention. These treatment modalities allowed patients to accustom to orthodontic treatment, from simple to a much more complex treatment. Usage of these appliances has no interference in the day-to-day activities of patients such as mastication, speech, and oral comfort. There are also no major issues related to appliance dislodgement, treatment compliance, and loss of appliance since these are fixed appliances.

Another appliance used in these described cases was the thermoplastic heat compressed Essix appliance. Essix appliance is postorthodontic treatment retainer, also known as vacuum-formed retainer. However, in our cases, the Essix appliance was used to open up the anterior bite to allow orthodontic movement of teeth. The Essix appliance also allows even distribution of the occlusal forces on the posterior teeth. The appliance provides an alternative to the use of glass ionomer cement (GIC) to raise open the anterior bite. Glass ionomer cement abrades fast and needs regular top-up to keep the bite open. Further, heavy occlusal forces may befall on the teeth placed with GIC and cause traumatic occlusion.

## 4. Conclusion

These cases showed that both the sectional orthodontic wire appliance and the 2 × 4 orthodontic appliance are viable appliances that can correct malposition teeth during mixed dentition stage when other removable appliance methods cannot perform the required task. Knowledge on the use of these appliances is handy in the dental care of children.

## Data Availability

None.

## References

[1] Dave B. H., Shah V. U., Bargale S., Deshpande A., Patel R., Sura S. (2018). Evaluation of parental perception regarding smile of children according to visual analog scale. *Journal of Integrated Health Sciences*.

[2] Vale T., Santos P., Moreira J., Manzanares M. C., Ustrell J. M. (2009). Perception of dental aesthetics in paediatric dentistry. *European Journal of Paediatric Dentistry*.

[3] Proffit W. R. (2019). On the aetiology of malocclusion. *British Journal of Orthodontics*.

[4] Houston W. J. B., Stephens C. D., Tulley W. J. (1992). *A Textbook of Orthodontics*.

[5] McDonald F., Ireland A. J. (1998). *Diagnosis of the Orthodontic Patient*.

[6] Zou J., Meng M., Law C. S., Rao Y., Zhou X. (2018). Common dental diseases in children and malocclusion. *International Journal of Oral Science*.

[7] Prakash P., Durgesh B. H. (2011). Anterior crossbite correction in early mixed dentition period using Catlan’s appliance: a case report. *ISRN Dentistry*.

[8] Borrie F., Bearn D. (2014). Early correction of anterior crossbites: a systematic review. *Journal of Orthodontics*.

[9] Wiedel A. P., Bondemark L. (2015). Stability of anterior crossbite correction: a randomized controlled trial with a 2-year follow-up. *The Angle Orthodontics*.

[10] Noar J. (2014). *Interceptive Orthodontics, a Practical Guide*.

[11] Yassen S. M., Naik S., Uloopi K. S. (2011). Ectopic eruption – a review and case report. *Contemporary Clinical Dentistry*.

[12] Consoli G., Luzzi V., Ierardo G., Sfasciotti G. L., Polimeni A. (2013). Occlusal trauma in mixed dentition: literature review. *European Journal of Paediatric Dentistry*.

[13] Carlos E. J., Diana B.-L. (2019). Direct anterior tracks: early and functional management of class III malocclusions—case report and literature review. *Case Reports in Dentistry*.

[14] Kolawole K. A., Folayan M. O. (2019). Association between malocclusion, caries and oral hygiene in children 6 to 12 years old resident in suburban Nigeria. *BMC Oral Health*.

[15] February 2020, https://orthodonticsaustralia.org.au/benefits-of-early-treatment/accessed

[16] İlisulu C., Uz S., Koruyucu M., Seymen F. (2019). Early interceptive orthodontic treatments: case series. *International Journal of Medical Investigation*.

[17] Millett D. T., Day P. (2016). *Clinical Problem Solving in Dentistry; Orthodontics and Paediatric Dentistry*.

[18] Sockalingam S. N. M. P., Khan K. A. M., Kuppusamy E. (2018). Interceptive correction of anterior crossbite using short-span wire-fixed orthodontic appliance: a report of three cases. *Case Reports in Dentistry*.

[19] Dowsing P., Sandler P. J. (2014). How to effectively use a 2 X 4 appliance. *Journal of Orthodontics*.

[20] Skeggs R. M., Sandler P. J. (2002). Rapid correction of anterior crossbite using a fixed appliance: a case report. *Dental Update*.

